# A Novel Method for Correcting Pelvic Tilt on Anteroposterior Pelvic Radiographs

**DOI:** 10.7759/cureus.6274

**Published:** 2019-12-02

**Authors:** Jeffrey M Muir, John Vincent, Joseph Schipper, Varsha D Gobin, Meinusha Govindarajan, Karlina Fiaes, Jonathan Vigdorchik

**Affiliations:** 1 Clinical Research, Intellijoint Surgical, Waterloo, CAN; 2 Medicine, Faculty of Applied Health Sciences, School of Public Health and Health Systems, University of Waterloo, Waterloo, CAN; 3 Orthopaedics, Research & Development, Intellijoint Surgical, Kitchener, CAN; 4 Psychology, University of Waterloo, Waterloo, CAN; 5 Epidemiology and Public Health, School of Public Health and Health Systems, University of Waterloo, Waterloo, CAN; 6 Orthopaedic Surgery, Hospital for Special Surgery, New York, USA

**Keywords:** hip arthroplasty, pelvic tilt, radiographs, imaging

## Abstract

Background

Anteroposterior (AP) pelvic radiographs remain the standard for pre- and postoperative imaging during total hip arthroplasty (THA), despite the known limitation of plain films, including the inability to adequately account for distortion caused by variations in pelvic orientation such as pelvic tilt. The purpose of this study was to develop a reliable method for correcting pelvic tilt on AP pelvic radiographs in patients undergoing THA.

Methods

CT scans from 20 patients/cadaver specimens (10 male, 10 female) were used to create 3D renderings, from which synthetic radiographs of each pelvis were generated. For each pelvis, 13 synthetic radiographs were generated, showing the pelvis at between -30° and 30° of pelvic tilt, in 5° increments. On each image, eight unique parameters/distances were measured to determine the most appropriate parameters for the calculation of pelvic tilt. The most reliable and accurate of these parameters was determined via regression analysis and used to create gender-specific nomograms from which pelvic tilt measurements could be calculated. The accuracy and reliability of the nomograms and correction method were subsequently validated using both synthetic radiographs (n=50) and stereoradiographic images (n=58).

Results

Of the eight parameters measured, the vertical distance between the superior margin of the pubic symphysis and the transischial line (PSTI) was determined to be the most reliable (r=-0.96, ICC=0.94). Using that parameter and applying the associated nomograms to 50 synthetic radiographs of random pelvic tilt, the mean difference between the actual pelvic tilt and that calculated using the correction method was 0.1°±5.1° (p=0.98, r=0.96). In 58 stereoradiographic images, the mean difference between actual and measured pelvic tilt was -0.2°±6.4° (p=0.74, r=0.77). The pooled results indicate no significant difference between actual (2.2°±13.9°) and measured pelvic tilt (2.1°±14.3°, p=0.93, r=0.91). No significant differences were noted based on gender.

Conclusions

Our method of correcting for pelvic tilt using the vertical distance from the pubic symphysis to the transischial line provides a reliable method for correcting for pelvic tilt on AP pelvic radiographs.

## Introduction

Anteroposterior (AP) pelvic radiographs remain the standard of care imaging modality for pre- and postoperative assessment during total hip arthroplasty (THA) [1-2], due largely to their ease of use, cost-effectiveness, and widespread availability [3-5]. Despite their ongoing use, radiographs are subject to artifacts and are limited by an inability to adequately account for the distortion caused by 3D variations of pelvic orientation such as pelvic tilt, obliquity, and rotation [3,6]. By some estimates, the likelihood of an AP pelvic view being free of artifact or distortion is only 30% [7], a point that illustrates the existing limitations of radiographs, which provide significantly less accurate cup position measurements when compared to other modalities such as CT imaging. The impact of this potential error can have a significant impact on post-THA image analysis, increasing the likelihood of errors in cup placement and leading to postoperative complications such as accelerated component wear, component loosening, reduced range of motion, pain, and a greater risk of impingement or dislocation [4,6,8].

Of specific concern to the THA surgeon is the impact of pelvic tilt (deviation of the anterior pelvic plane (APP) from the coronal plane), as an undetected tilt may contribute to the inaccurate evaluation of cup placement [9-13]. Increasing anterior or posterior pelvic tilt is known to introduce errors in the measurement of acetabular orientation [3,5-6,11], especially anteversion, which is highly susceptible to changes in pelvic tilt [1,2,8,12], with each degree change in pelvic tilt known to alter anteversion by 0.77-0.80° [14-15]. Various methods of evaluating and correcting pelvic tilt on radiographs have been proposed [3,16-18]; however, none have proven accurate and reliable. Some proposed methods have been found to be inappropriate for a THA population [19] while others require additional data or imaging such as standing lateral radiographs [20], which are not common practice following THA. Currently, there is a lack of consensus regarding a widely accepted and clinically validated method of tilt correction.

Considering the significant implications of inaccurate acetabular cup position due to imaging errors such as pelvic tilt, a reliable method for the correction of pelvic tilt on radiographs is required. The objective of this study was to develop and test a method for accurately measuring pelvic tilt on anteroposterior (AP) radiographs. Our hypothesis was that this method would prove accurate when validated against synthetic and stereoradiographic images with known pelvic tilt.

## Materials and methods

For the purposes of this study, “pelvic tilt” was defined as the angle of the anterior pelvic plane (APP) relative to a vertical axis (APP tilt: APPt). As such, pelvic tilt (APPt) of 0° represents a pelvis with the ASIS and anterior border of the symphysis pubis being oriented vertically. Anterior or positive tilt reflects the movement of the ASIS in an anterior direction relative to the pubic symphysis; posterior or negative tilt reflects the posterior movement of the ASIS.

Imaging

This study used synthetic radiographic images created from segmented CT scans and 3D stereoradiographic clinical imaging (EOS) to create and validate our correction method. Ethics approval for the use of patient images was received from the participating institution prior to data collection. 

CT Scans

**Figure 1:**
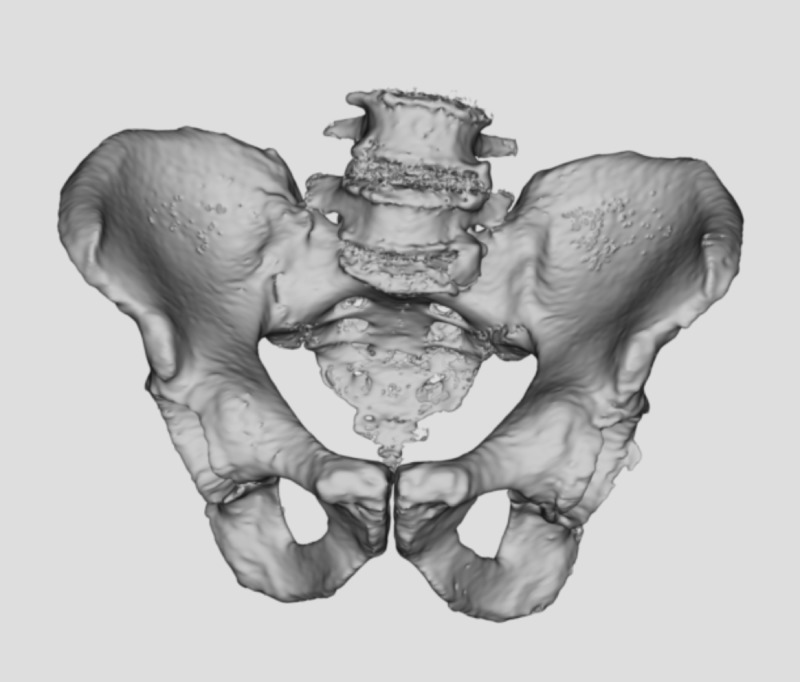
3D rendering created from CT scan

The CT scans of six cadavers (one female, five male) used in previous THA studies [21-23] and 14 patients (nine female, five male) undergoing primary THA were obtained using a GE LightSpeed™ 16 imager (GE Healthcare, Chicago, Illinois; 140 kV, 600 mA at 0.8 sec revolution time and 0.625 mm slice thickness). Each CT scan was segmented using medical image computing software (3-D Slicer, version 4.6 [24]). Segmented CT scans were then used to create 3-D renderings of each pelvis (Figure 1).

Synthetic Radiographs

**Figure 2:**
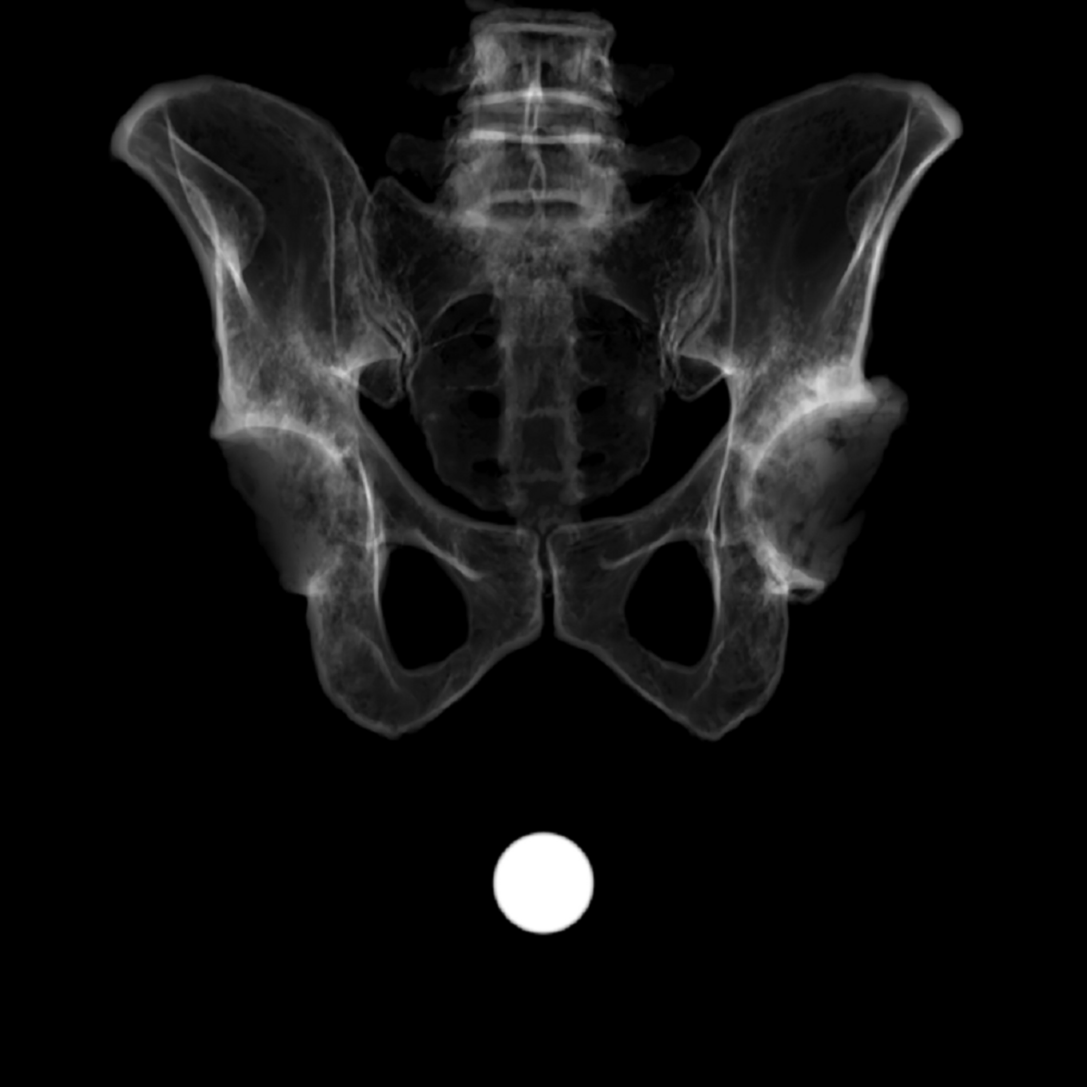
Synthetic radiograph created from 3D rendering; the 25.4 mm scaling ball is visible in the image

Each 3D rendering (n=20: 10 male, 10 female) was used to create a geometrically accurate, simulated AP radiograph, using the imaging simulator XRaySim [25] (Figure 2). The imaging simulator further allows the user to alter the orientation of the image without distorting the dimensions or spatial relationships. Thus, we were able to create a series of synthetic AP radiographs at specific pelvic tilts, from which measurements could be obtained. For each pelvis, 13 distinct synthetic images were created, showing the pelvis oriented at between -30° and +30° of tilt, in 5° increments. Synthetic images were created by an author (JS) not involved in the measurement of pelvic tilt parameters.

Stereoradiographic Imaging

Stereoradiograhic imaging (EOS Imaging, SA, Paris, France) from 58 patients undergoing primary or revision THA (none of whom were part of the cohort used to create the 3D renderings) were obtained and used to validate our correction method. Each EOS image consists of perpendicular AP and lateral full-body radiographic views, taken simultaneously. As such, the pelvic tilt (APPt) measured from the lateral view matches that of the AP view.

Part I: Evaluation of pelvic tilt measurements

**Figure 3:**
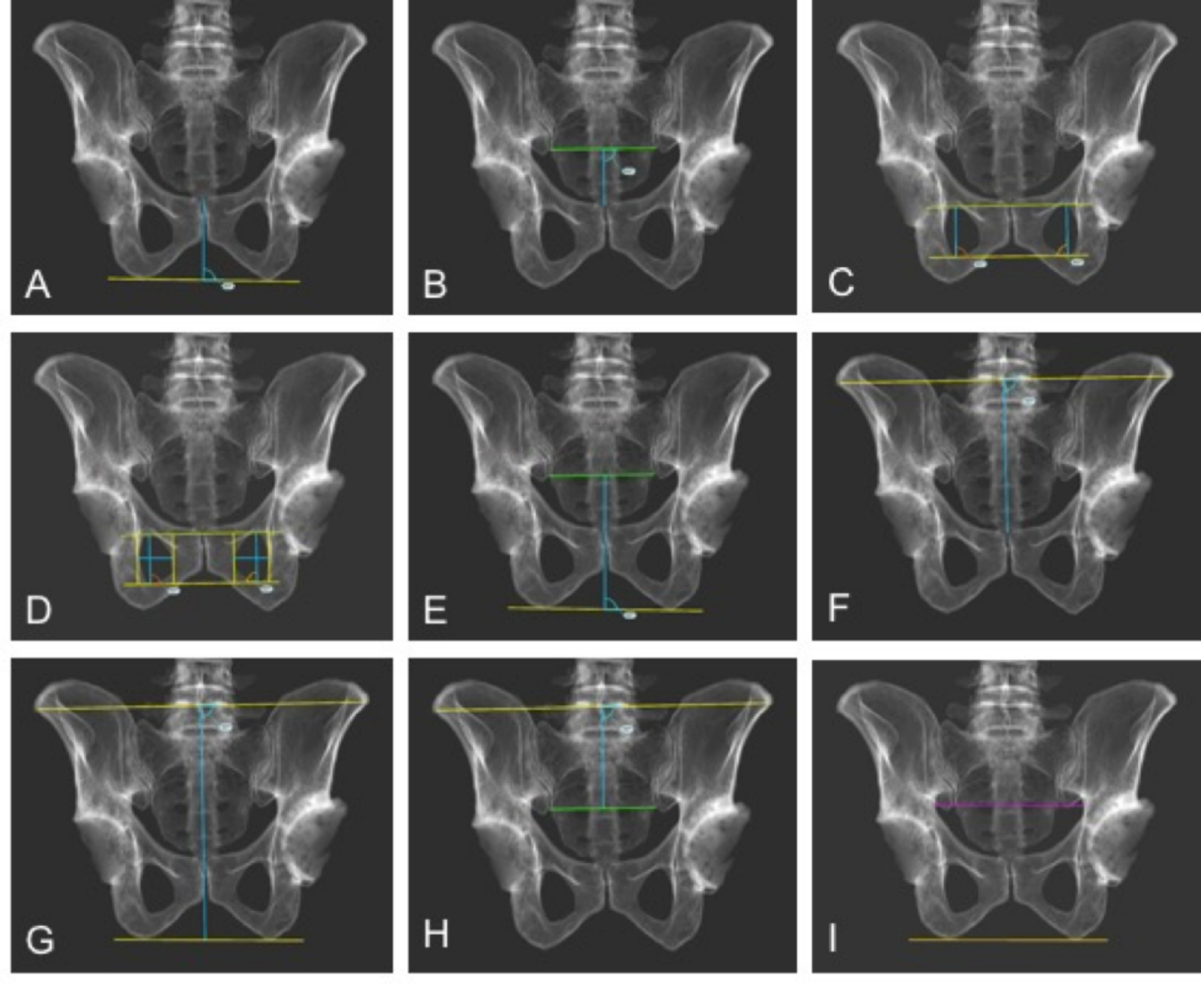
Pelvic measurements tested included the vertical distance from the transischial line to the upper border of the pubic symphysis (PSTI, A); the vertical distance from the pubic symphysis to a line intersecting the lower borders of the sacroiliac joints (trans-SI line) (PSSI, B); the height of the obturator foramen (C); the height and width of the obturator foramen (D); the vertical distance from the transischial line to the trans-SI line (TISI, E); the vertical distance from the pubic symphysis to the trans-ASIS line (PSTA, F); the vertical distance from the transischial line to the trans-ASIS line (TITA, G); and the vertical distance from the trans-SI line to the trans-ASIS line (SITA, H). To help scale each radiograph, the pelvic outlet distance (POD) was also measured as the longest horizontal distance across the pelvic outlet, parallel to the transischial line (I). Borders or reference lines are shown in yellow; the trans-sacroiliac joint line is shown in green; measured distances are shown in blue; the pelvic outlet distance is shown in purple.

Eight measurement parameters that incorporated landmarks that are uniformly visible on radiographs were identified and measured on each synthetic image. Specifically, the following distances were measured on synthetic AP pelvic radiographs (Figure 3):

A. the vertical distance from the transischial line to the upper border of the pubic symphysis (PSTI),

B. the vertical distance from the pubic symphysis to a line intersecting the lower borders of the sacroiliac joints (trans-SI line) (PSSI),

C. the height of the obturator foramen,

D. the height and width of the obturator foramen,

E. the vertical distance from the transischial line to the trans-SI line (TISI),

F. the vertical distance from the pubic symphysis to the trans-ASIS line (PSTA),

G. the vertical distance from the transischial line to the trans-ASIS line (TITA), and

H. the vertical distance from the trans-SI line to the trans-ASIS line (TSITA).

**Figure 4:**
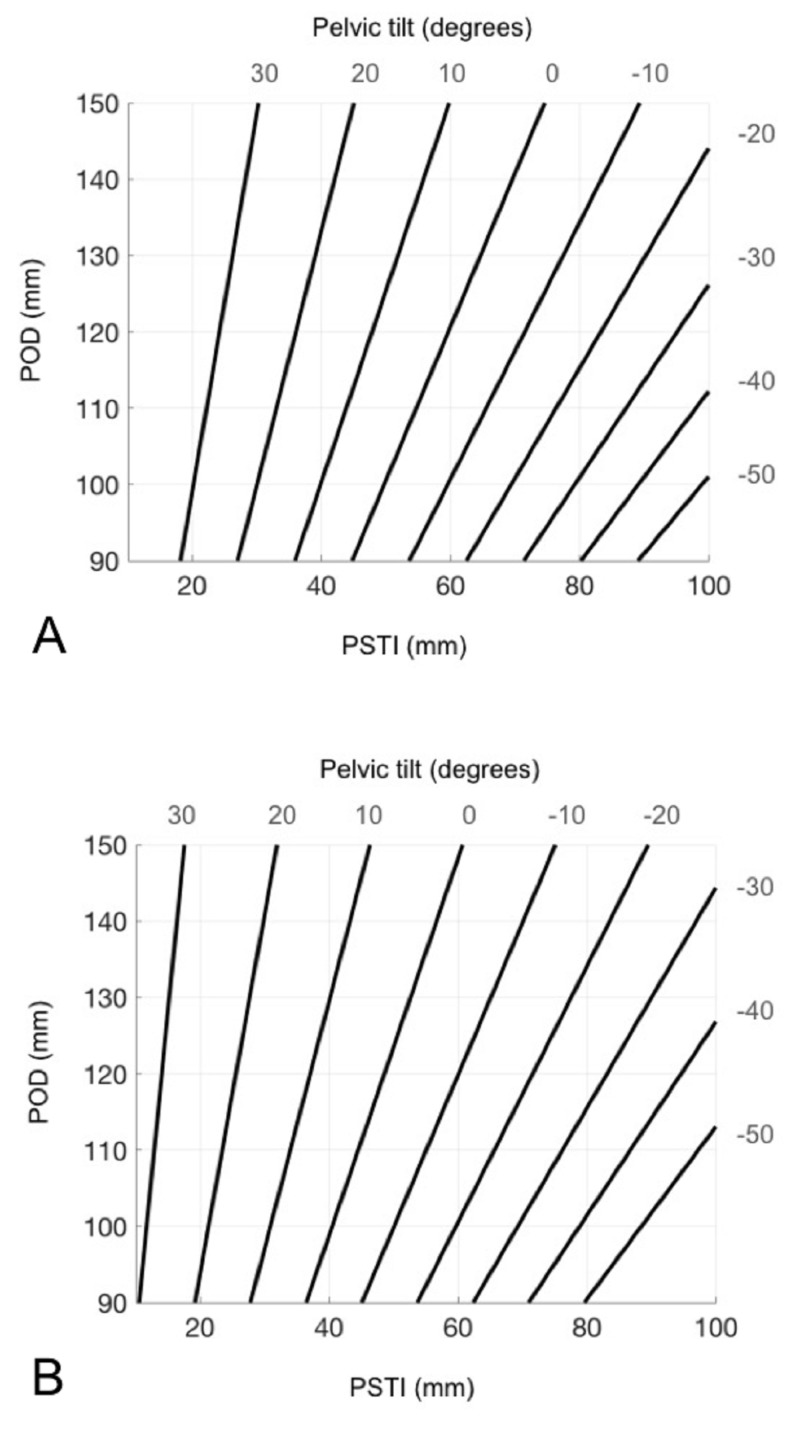
Nomograms were created for male (A) and female (B) pelvises based on measurements from synthetic radiographs. Tilt is calculated based on the intersection of the POD and PSTI measurements. E.g. on a pelvic radiograph of a female pelvis with a POD of 120 mm and a PSTI of 40 mm, +7.5° of APPt would be present. POD: pelvic outlet distance; PSTI: the vertical distance from the transischial line to the upper border of the pubic symphysis; APPt: anterior pelvic plane tilt

To assist with the scaling of the images, we also measured the maximum horizontal distance of the pelvic outlet, along a line parallel to the transischial line (POD measurement). All measurements were completed in triplicate and the values averaged to create a set of measurements for each specific synthetic AP radiograph. All radiographic measurement was completed using TraumaCad (version 2.5) software (Brainlab, Chicago, IL). The relationships were plotted and an equation of each line calculated, from which gender-specific nomograms could be created (Figure 4). 

Part II: Validation of tilt-correction nomograms

Once the spatial relationships had been established and the nomograms created, their accuracy in measuring APPt from AP images was validated using a combination of synthetic radiographs and EOS images.

Synthetic AP Radiographs

The initial validation of tilt correction was accomplished using synthetic AP radiographic images. From the 20 pelvises used in this study, 10 were chosen at random, from which 50 synthetic images (five from each pelvis) were created at random orientations between -30° and +30° of pelvic tilt. Test images were created by an author (JS) not involved in the measurement of parameters for pelvic tilt. A random number generator (www.random.org) was used to ensure the randomization of image selection and tilt orientation.

The PSTI and POD distances were measured from each of these 50 images by reviewers blinded to the actual APPt. Measurements for each image were completed in triplicate and averaged to arrive at final PSTI and POD measurements. Averaged measurements were then applied to our nomograms to calculate the APPt for each AP image. The calculated tilt was then compared with the actual APPt for each image.

Stereoradiographic Images

Clinical validation of the tilt-correction calculations used 58 EOS images from patients undergoing primary or revision THA. On the AP EOS image, the PSTI and POD distances were measured by reviewers blinded to the true APPt. All measurements were made in triplicate and the results averaged to arrive at final values. The resulting measurements from each image were then applied to the nomograms to calculate the APPt from the AP image. On the lateral EOS images, APPt was measured as the angle between a line parallel to the vertical edge of the image and a line intersecting the anterior superior iliac spine (ASIS) and the pubic symphysis. Again, measurements were made in triplicate and averaged. The difference between tilt calculated from the AP view and nomograms and the actual value measured from the lateral view was then calculated.

Statistical analysis

Alpha was set a priori to p<0.05 for all statistical comparisons between the radiographic and navigation data. Intra-observer and radiographic parameter reliability were assessed using the intra-class correlation coefficient (ICC). Mean values are expressed as mean (standard deviation) and compared using the mean differences and Student’s t-test or single-factor ANOVA as appropriate. Correlations were evaluated using Pearson’s r or logistic regression analysis.

## Results

Part I: Evaluation of pelvic tilt measurements

The evaluation of the various measurement parameters is summarized in Table 1. While several parameters demonstrated satisfactory correlations when tested using logistic regression analysis, the highest reliability with respect to the consistency of measurement were the PSTI (symphysis-transischial), PSSI (symphysis-SI joint), TISI (SI joint-transischial), and OFH (obturator height) measurements. Of these four parameters, distances were unable to be accurately measured in 5/65 (7.7%) of cases in the PSSI and TISI parameters and in 11/65 (16.9%) of cases in the case of the OFH. Using the PSTI parameter, 100% of measurements were accurately made. Based on this combined analysis, we chose to proceed with only the PSTI distance, as it was shown to be the most reliable and repeatable.

**Table 1:** Summary of parameters measured 1. Multiple logistic regression 2. Measurements missed due to an inability to adequately identify required landmarks on the radiograph ICC: intraclass correlation; PSTI: pubic symphysis to transischial line; PSSI: pubic symphysis to sacroiliac joint line; OFH: obturator foramen height; OFR: obturator foramen height:width ratio; TISI: transischial line to sacroiliac joint line; PSTA: pubic symphysis to trans-ASIS line; TITA: transischial line to trans-ASIS line; SITA: sacroiliac joint to trans-ASIS line

Parameter	r^1^	Standard deviation	p-value	ICC	Missed measurements^2^
PSTI	-0.96	0.46	<0.001	0.94	0
PSSI	0.96	0.98	<0.001	0.87	5/65 (7.7%)
OFH	0.94	0.24	<0.001	0.85	11/65 (16.9%)
OFR	0.91	0.02	<0.001	0.75	12/65 (18.5%)
TISI	0.83	0.92	<0.001	0.52	5/65 (7.7%)
PSTA	0.10	1.74	0.41	0.14	0
TITA	-0.83	1.77	<0.001	0.68	0
SITA	0.93	2.04	<0.001	0.84	5/65 (7.7%)

Measurement of the PSTI and POD distances allowed for the creation of equations representing the relationship between these two parameters and pelvic tilt for each of the male and female pelvises measured. Gender-specific nomograms were created from the equations to provide a tool for calculation of pelvic tilt (Figures 4A-4B).

Part II: Validation of tilt-correction nomograms

Synthetic Radiographs

In the 50 synthetic radiographs used for validation, there was no statistically significant difference between the mean APPt measured using the correction method (0.6°±18.6°) and the actual tilt (0.5°±17.9°, p=0.98). The mean difference between the measured and actual APPt was 0.1°±5.1° (range: -8°-14°), with 90% (45/50) of differences less than 5°. The absolute mean difference between measurement methods was 3.7°±3.5°, with 80% (40/50) of differences <5°. Measured tilt correlated very strongly with tilt measurements calculated from the synthetic AP images (r=0.96). The PSTI-POD calculation correctly predicted the direction of APPt in 100% of cases (Table 2). 

**Table 2:** Sensitivity analysis of APPt measurements calculated from synthetic radiographs 1. Correlation between actual and measured APPt values 2. T-test comparing mean of actual values vs. mean of measured values

	Actual	Measured	Delta	Absolute delta	Pearson’s r^1^
All, mean (SD), n=50	0.5 (17.9)	0.6 (18.6)	0.1 (5.1)	3.7 (3.5)	0.96
Non-multiples of 5, mean (SD), n=39	0.2 (18.0)	0.4 (18.9)	0.3 (5.4)	3.7 (3.8)	0.96
p-value^2^	0.92	0.96	0.90	0.996	-

A subgroup analysis based on gender found no significant differences between males and females. The correlations between actual and measured APPt remained very strong in both male (r=0.97) and female (r=0.98) pelvises. There was no statistically significant difference between actual and measured tilt in either the males (-1.8°±17.5° vs. 0.8°±19.6°, p=0.64) or female (2.8°±18.3° vs. 0.4°±18.0°, p=0.62) pelvises. In both male and female pelvises, 80% (20/25 for each of) differences were <5° (Table 3).

**Table 3:** Gender-specific sensitivity analysis of APPt measurements calculated from synthetic radiographs 1. Correlation between actual and measured APPt values 2. T-test comparing mean of actual values vs. mean of measured values APPt: anterior pelvic plane tilt

	Actual	Measured	Delta	Absolute delta	Pearson’s r^1^
Male, mean (SD) All (n=25) Non-mx of 5 (n=22)	-1.8 (17.5) -1.3 (17.0)	0.8 (19.6) 1.7 (19.0)	2.6 (5.1) 3.0 (5.2)	3.4 (4.6) 3.5 (4.8)	0.97 0.96
p-value	0.93	0.88	0.79	0.92	-
Female, mean (SD) All (n=25) Non-mx of 5 (n=17)	2.8 (18.3) 2.1 (19.4)	0.4 (18.0) -1.2 (19.3)	-2.4 (3.9) -3.3 (3.1)	4.1 (1.9) 4.0 (2.1)	0.98 0.99
p-value^2^	0.90	0.78	0.41	0.90	-

Of the 50 random orientations used for validation, 11 were multiples of 5, i.e. the same orientations used to create the initial equations and nomograms. Although the nomograms were created by pooling data from 260 unique images and not from one single image or pelvis, we nonetheless performed a sensitivity analysis, comparing the actual and measured tilt values in only those 39 synthetic images that were not multiples of 5. We found no statistically significant differences between the findings in the entire group of 50 images and the subgroup of 39 images (Table 2). Further subgrouping of this cohort of 39 patients, based on gender, likewise found no significant differences between mean tilt measurements, mean differences between measurements, or proportion of differences <5° in the cohort of 39 when compared to the full cohort of 50 (Table 3).

Stereoradiographic Images

In the 58 EOS images used to validate the correction method in clinical imaging, the mean APPt calculated from AP EOS images (3.8°±8.2°) did not differ significantly from that measured from the lateral EOS images (3.2°±9.9°, p=0.74). Tilt measured from AP images differed by -0.2°±6.4° when compared with values measured from lateral images (absolute mean difference: 5.3°±3.5°). A strong correlation (r=0.77) was noted between the tilt measured from AP and lateral images. 

The PSTI-POD measurements correctly predicted the direction of tilt in 70% of cases (75% in females, 66% in males). In cases where the APPt was greater than 5°, the PSTI-POD measurement correctly predicted the direction of tilt in 98% of cases (100% (19/19) in females and 95% (20/21) in males. 

Calculations from AP images were more accurate in predicting tilt in females than males (females: r=0.83, males: r=0.69), although the average differences between calculated and measured tilt were not significantly different between genders (females: 0.59°±6.1° vs. males: -0.71°±6.6°, p=0.45). Likewise, the absolute mean differences between calculated and measured pelvic tilt were not significantly different between genders (females: 4.9°±3.6° vs. males: 5.6°±3.5°, p=0.48).

Pooled Analysis

Combining all results from the synthetic and EOS images, the tilt measured from AP images (2.1°±14.3°) was not significantly different from the actual tilt (2.2°±13.9°, p=0.92). Eighty-two percent (89/108) of differences were <5° in the pooled analysis and a very strong correlation was noted between actual and measured tilt (r=0.91). Gender did not affect the pooled analysis. Correlations for both remained very strong (male: 0.90, female: 0.94) and there was no significant difference between the mean differences between actual and calculated tilt in males vs. females (0.11°±6.7° vs. -0.96°±5.2°, p<0.05).

## Discussion

Postoperative AP radiographs remain the clinical standard for the evaluation of component positioning after THA, despite well-known inaccuracies associated with patient positioning, including pelvic tilt. No reliable methods currently exist to correct for APPt on AP radiographs, introducing a potentially significant amount of error into the image and any measurements, such as cup position, taken from those images. To address this lack of reliable correction methods, we used segmented CT scans of patients undergoing total hip arthroplasty to create synthetic AP radiographs and develop a method for correcting for APPt. We verified our method on both synthetic images and EOS images of patients undergoing THA. We observed a high level of correlation between actual and measured tilt, suggesting our correction method may be viable for this patient population.

Anterior pelvic plane tilt represents a significant contributor to inaccuracies in cup position during THA. Studies have shown that for each degree of tilt, anteversion is altered by up to 0.80° [14-15]. Combined with the inherent inaccuracies associated with radiographic imaging, the potential impact of unaccounted-for APPt is substantial. Several attempts have been made to develop a method for correcting for pelvic malpositioning on radiographs, with limited success [3,6,10,26]. We previously evaluated the ability of one commonly cited correction method [3] and found that it was unable to accurately estimate tilt in a population of patients undergoing THA [19]. The authors of that study used a younger patient population and evaluated several methods for estimating tilt. They concluded that the measurement of the vertical distance between the pubic symphysis and the sacrococcygeal joint was the most accurate estimator; however, we noted that the sacrococcygeal joint was visible in only a small proportion of cases, making this method difficult to apply broadly. Additionally, other proposed methods of estimating tilt require additional input or measurement, such as lateral views [20] or additional data on the angle of the central beam of the X-ray [27], information that may be difficult to obtain and/or is beyond the current standard of care. In our study, we chose landmarks that are much more likely to be visible on standard AP radiographs, i.e. landmarks not obscured by bowel gas or other abdominal artifacts, and not subject to poor focus due to variations in focal distance settings during radiography. Not only were the required landmarks visible in all cases in our study, but no additional input was required to complete our correction. Also, the additional measurement of the horizontal pelvic outlet distance to the vertical PSTI distance provided an important scaling factor to increase the accuracy of measurements. By measuring perpendicular distances, we were able to not only estimate tilt but to scale the image in two dimensions, thus removing any distortion created by changes in focal distance.

A key aspect of our study as compared with previous studies was the use of synthetic radiographs and stereoradiographic (EOS) images to validate our calculations. The use of the synthetic images in the creation of our nomograms greatly increased the accuracy with which we measured the PSTI and POD distances. Previous studies relied on AP and lateral images to provide data regarding actual pelvic tilt [3,6,20]. As such, the actual tilt for each image was required to be measured from the lateral image, which thus did not eliminate the inherent distortion or inaccuracy present on radiographic imaging. As well, using traditional standing AP and lateral radiographs requires the patient to change position between exposures, which could introduce a source of error, as there is no way to ensure that their pelvic tilt remains consistent in both images. Conversely, we created synthetic radiographic images from segmented CT scans, providing a radiographic image with increased accuracy, as each was based on a detailed CT scan. Also, as the XRaySim software allowed us to establish the orientation of each image exactly, we created images at specific and exact tilt values, without any error or distortion. The resulting APPt values were thus calculated with fewer areas for error, increasing the accuracy of our study, a fact that is reflected in our observed correlation in synthetic images (0.96). Our study is further strengthened by the use of clinically relevant imaging to confirm our calculations in patients. The use of EOS imaging provides improved accuracy over radiographs, as the images are taken simultaneously, thus removing the potential error associated with patient movement. The resulting strong correlation (0.77) reflects this minimal imaging error. Indeed, the use of the sacrococcygeal joint as a landmark on plain film radiographs resulted in a correlation of only 0.68 [3]. Our use of EOS imaging eliminates the error associated with the image itself and better reflects the error associated with the calculation method itself.

This study has limitations. The use of synthetic radiographs may raise concerns regarding the accuracy and/or clinical relevance of this imaging modality. However, all software used to create the 3D renderings of CT scans and the synthetic radiographs themselves are widely available and regularly used for such studies [28-30]. Also, CT segmenting, synthetic image generation, and all image analyses were performed by different study team members, to minimize sources of bias regarding image analysis. As well, the use of EOS images enhanced our analysis, as it provided clinically relevant images from which we were able to extract accurate measurements for comparison.

## Conclusions

Using synthetic radiographs and 3D EOS images from patients undergoing THA, we calculated the relationship between the vertical distance from the transischial line to the pubic symphysis and horizontally across the pelvic outlet and created a method to estimate the degree of tilt of the APP on AP radiographs. We validated our method using both synthetic radiographs and EOS images and found that it represents a reliable and verified method for estimating APPt on AP radiographs.
